# Long‐Term Treatment With Emapalumab in an Adult Patient With Refractory Hemophagocytic Lymphohistiocytosis and Systemic Lupus Erythematosus: A Case Report

**DOI:** 10.1155/crh/7722625

**Published:** 2026-07-09

**Authors:** Hind Salama, Ghada Alshehri, Ayel Yahya, Areej Almugairi

**Affiliations:** ^1^ Department of Oncology, Division of Adult Hematology, King Abdulaziz Medical City, Riyadh, Saudi Arabia, ngha.med.sa; ^2^ College of Medicine, King Saud Bin Abdulaziz University, Riyadh, Saudi Arabia, ksau-hs.edu.sa; ^3^ King Abdullah Medical Research Center, Riyadh, Saudi Arabia; ^4^ Department of Pathology and Laboratory Medicine, King Abdulaziz Medical City, Riyadh, Saudi Arabia, ngha.med.sa

**Keywords:** case report, emapalumab, hemophagocytic lymphohistiocytosis, macrophage-activation syndrome, myelodysplastic syndrome, systemic lupus erythematosus

## Abstract

**Background:**

Hemophagocytic lymphohistiocytosis (HLH) is a life‐threatening hyperinflammatory syndrome characterized by excessive immune activation due to persistently activated cytotoxic lymphocytes and macrophages. HLH can lead to multiorgan dysfunction and can be fatal if left untreated. HLH secondary to autoimmune diseases is referred to as macrophage activation syndrome (MAS).

**Case Presentation:**

A female Arab Saudi patient in her late 20s with a history of systemic lupus erythematosus (SLE) presented with reduced oral intake, abdominal pain, decreased urine output, tachypnea, shortness of breath, and acute heart failure. MAS secondary to SLE and associated stress cardiomyopathy was suspected. Despite initial treatment with the HLH‐94 protocol, the patient showed no improvement and developed Gram‐negative septicemia with worsened cytopenia. Emapalumab, a monoclonal antibody that targets interferon gamma (IFN‐γ), was introduced as a salvage therapy, resulting in rapid clinical and biochemical improvement, including normalization of cardiac function. The patient received emapalumab for around 17 months, with good tolerability apart from a brief period of CMV reactivation, achieving sustained remission. Following discontinuation, HLH recurred in the context of newly diagnosed myelodysplastic syndrome and was refractory to conventional therapy but responded again to emapalumab retreatment. At the time of last follow‐up, the patient remained clinically stable with no evidence of active HLH while continuing emapalumab therapy in the context of ongoing treatment for her underlying conditions.

**Conclusions:**

This case report highlights the potential of emapalumab as an effective therapeutic option for the treatment of relapsed/refractory HLH in adults, including in SLE‐ and malignancy‐associated disease not responding to conventional therapy. It supports its role in achieving rapid disease control, enabling prolonged remission with extended use, and allowing successful retreatment in recurrent HLH, including in high‐risk settings with poor outcomes such as malignancy‐associated HLH.

## 1. Background

Hemophagocytic lymphohistiocytosis (HLH) is a life‐threatening hyperinflammatory syndrome driven by uncontrolled activation of cytotoxic lymphocytes and macrophages, resulting in multiorgan dysfunction and high mortality if left untreated [1]. Clinically, HLH presents with nonspecific features including persistent fever, cytopenias, hyperferritinemia, and organ dysfunction [1].

HLH may be primary (familial/genetic) or secondary to triggers such as infection, malignancy, or autoimmune disease [2, 3]. When associated with autoimmune conditions, it is referred to as macrophage‐activation syndrome (MAS), a subtype of secondary HLH characterized by similar clinical and immunopathological features [4]. The development of MAS secondary to systemic lupus erythematosus (SLE), one of the major causes of MAS in adults, is linked to disease activity and concurrent infections, often requiring intensive care and resulting in death [2–10].

Current treatment strategies for HLH are largely extrapolated from pediatric protocols, most notably the HLH‐94 regimen, which combines glucocorticosteroids (GCSs), etoposide, and cyclosporin A [6–8]. However, outcomes in adults with secondary HLH remain suboptimal due to delays in diagnosis, significant treatment‐related toxicity, and low survival rates [9–12]. These limitations have driven interest in targeted immunotherapies.

Emapalumab is a monoclonal antibody targeting interferon gamma (IFN‐γ), a central cytokine in HLH pathophysiology, with demonstrated efficacy in relapsed/refractory disease in both children and adults [13–15]. However, data in adults, particularly in MAS associated with SLE, remain limited, and evidence regarding prolonged use or retreatment is sparse.

This case report describes a critically ill adult patient with SLE‐associated MAS refractory to conventional therapy, successfully treated with prolonged emapalumab, including retreatment during relapse associated with malignancy, highlighting its potential role in complex adult HLH.

## 2. Case Presentation

A female Arab Saudi patient in her late 20s presented to the emergency department in October 2022 with reduced oral intake and abdominal pain. She was diagnosed with *Clostridioides difficile* infection and admitted for intravenous hydration and antibiotic therapy.

The patient had a known history of SLE, diagnosed in 2016 by her treating rheumatologist on the basis of autoimmune serology consistent with SLE, including antinuclear antibodies, anti‐SSA/SSB antibodies, and anti–double‐stranded DNA antibodies (225 IU/mL). Her disease course was complicated in 2021 by proliferative lupus glomerular nephritis, for which she was treated with mycophenolate mofetil, GCS (prednisolone; 10 mg daily), hydroxychloroquine, and belimumab.

Renal function continued to worsen despite therapy, and intravenous rituximab was subsequently initiated on a weekly basis. Following the second dose of rituximab, the patient developed rapid and severe clinical deterioration characterized with high‐grade fever, progressive cytopenia, and acute respiratory distress syndrome requiring intubation and vasopressor support. Her condition progressed to multiorgan dysfunction with escalating inotropic requirements. On examination, she was febrile (39.8°C), tachypneic, and hypoxic despite high‐flow oxygen therapy, with profound coma‐level neurological impairment (Glasgow Coma Scale score of 3/15). Transthoracic echocardiography revealed an acute reduction in left ventricular ejection fraction to 15%, compared with a baseline of > 55% 2 weeks earlier, suggestive of stress‐induced cardiomyopathy in the setting of severe systemic inflammation.

Cytokine release syndrome secondary to rituximab was initially considered by the referring rheumatology team, and tocilizumab was administered based on this working diagnosis. Blood cultures obtained at initial evaluation were negative. Given the absence of clinical improvement after three doses of tocilizumab and progressive deterioration, the patient was subsequently referred to the hematology team, at which point MAS secondary to SLE was strongly suspected as the primary unifying diagnosis.

**FIGURE 1:**
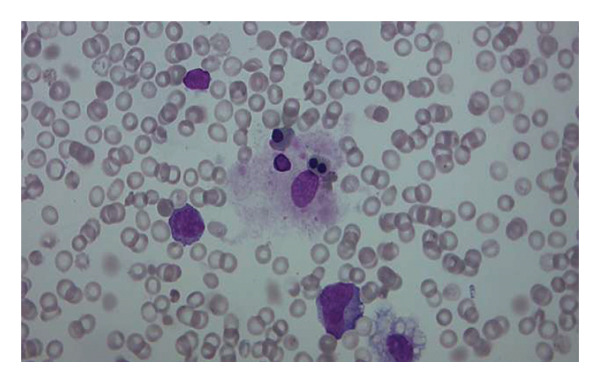
Bone marrow aspirate showing few histiocytes with evident hemophagocytosis to erythroid precursors (MGG stain, 60x magnification).

**FIGURE 2:**
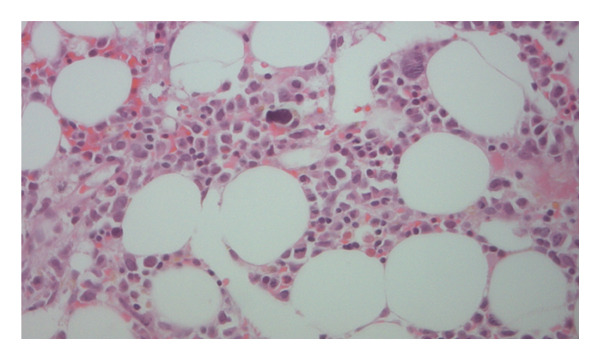
Bone marrow biopsy showing hypocellular bone marrow with histiocyte proliferation (H&E stain, 40x magnification).

A comprehensive diagnostic evaluation was then performed, including application of the HLH 2004 criteria, calculation of the H‐score, bone marrow biopsy, and genetic testing for familial HLH‐association mutations. Bone marrow biopsy demonstrated marked histiocytic activation with hemophagocytosis (Figures 1 and 2). Genetic testing excluded known pathogenic variants associated with primary HLH. In contrast, the patient fulfilled 6 of the 8 HLH‐2004 criteria, including persistent fever, pancytopenia, hyperferritinemia, hypertriglyceridemia, elevated interleukin‐2 (IL‐2) receptor levels, and bone marrow hemophagocytosis (Table 1). The H‐score was 269, corresponding to a > 99% probability of HLH. Based on the integration of clinical, laboratory, and histopathological findings, a diagnosis of MAS secondary to SLE was established. At the time of HLH diagnosis, SLE disease activity was high, with a SLE Disease Activity Index 2000 (SLEDAI‐2K) score of 11, driven by active vasculitis with limb ischemia, fever, leukopenia, and thrombocytopenia.

**TABLE 1:** Patient H‐score and HLH‐2004 criteria at diagnosis.

H‐score			HLH‐2004			
Criteria	Extent	Value	Criteria		Extent	Value
Known underlying immunosuppression	Yes	18				

Temperature (°C)	> 39.4	49	Fever		Yes	1

Organomegaly	No	0	Splenomegaly		No	0

Number of cytopenia defined as	Cytopenia in 3 lineages	34	Cytopenia (affecting ≥ 2 lineages in peripheral blood)		Yes	1
1. WBC ≤ 5 × 10^3^/mm^3^	1. WBC: 3.5 × 10^3^//mm^3^					
2. Hb ≤ 9.2 g/dL	2. Hb: 7 g/dL					
3. Platelet ≤ 110 × 10^3^/mm^3^	3. Platelet: 14 × 10^3^/mm^3^					

Ferritin (μg/L)	> 33,511	50	Ferritin ≥ 500 μg/L		Yes	1

Triglyceride (mmol/L)	4.19	64	Hypertriglyceridemia and or hypofibrinogenemia		Yes	1
Fibrinogen (g/L)	2.89	0				

AST (U/L)	248	19	Soluble CD25 (IL‐2 receptor) ≥ 2400 U/L		Yes 6400 U/L	1

Hemophagocytosis in bone marrow	Yes	35	Hemophagocytosis in bone marrow, spleen, liver, lymph nodes, or other tissues		Yes	1

			Low or absent natural killer (NK) cell activity		No	0

Total score		269	Total score		6/8	

Probability of HLH		> 99%				

Note: AST: aspartate aminotransferase; Hg: hemoglobin; HLH: hemophagocytic lymphohistiocytosis; IL‐2: interleukin‐2.

Abbreviation: WBC, white blood cell.

The patient was initially managed with the HLH‐94 protocol (dexamethasone, etoposide, and cyclosporin A). However, after two doses of etoposide, the patient developed *Pseudomonas aeruginosa* septicemia associated with worsening cytopenia and rising ferritin levels (> 35,000 μg/L), with no clinical improvement. She was treated with broad‐spectrum antibiotics (piperacillin–tazobactam) and intravenous immunoglobulin (1 g/kg for 2 days). Despite this, her HLH parameters did not improve, and escalating doses of inotropic support were required.

Given treatment‐related myelosuppression, superimposed etoposide‐linked septicemia, and progressive worsening of HLH parameters including rising ferritin levels, the HLH‐94 protocol was discontinued on 9 November 2022. Therapy was transitioned to emapalumab as salvage therapy and as a less myelosuppressive therapeutic alternative. As part of a comprehensive infection risk mitigation strategy, and in consultation with an infectious disease specialist, baseline screening for active and latent infections (CMV and EBV viremia by PCR and latent tuberculosis by QuantiFERON) was performed alongside initiation of prophylactic antiviral therapy. This approach aimed to address the risk of latent viral reactivation associated with emapalumab‐induced IFN‐γ blockade. Isoniazid prophylaxis was also commenced to mitigate the risk of latent tuberculosis reactivation in an endemic setting, together with *Pneumocystis* pneumonia and antifungal prophylaxis.

**FIGURE 3:**
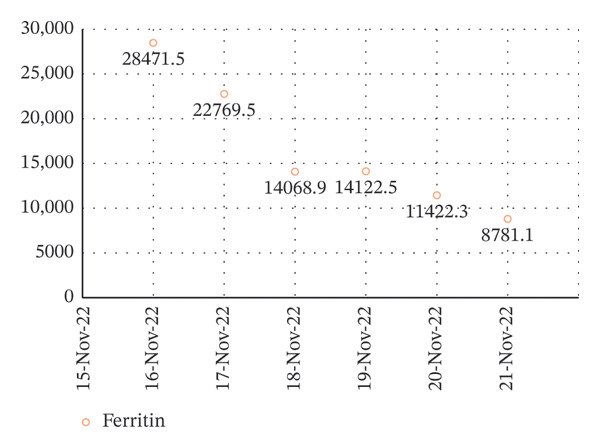
Progressive, rapid decrease in the serum ferritin concentration (μg/L) after emapalumab initiation.

Emapalumab was initiated at 1 mg/kg and escalated to 3 mg/kg. Following the third dose, the patient demonstrated marked clinical and biochemical improvement in HLH parameters (Figure 3). Ferritin and triglyceride levels remained stable on therapy, and the dose was increased to 6 mg/kg after 10 days, resulting in further improvement in inflammatory parameters, including ferritin (8781.1 μg/L), triglycerides (1.2 mmol/L), and IL‐2 receptor (1680 U/mL), and normalization of leukocyte and platelet counts (6.8/mm^3^ and 166,000/mm^3^, respectively). Hemoglobin remained low at 9 g/dL, likely due to anemia caused by chronic disease.

By Week 7 of emapalumab therapy, the patient was successfully weaned from mechanical ventilation and discharged from the intensive care unit. Repeat echocardiography revealed normalization of cardiac function. She was subsequently discharged from hospital, although she remained dialysis‐dependent due to end‐stage renal disease secondary to lupus nephritis, which was present prior to the HLH episode.

An attempt to introduce ruxolitinib in combination with emapalumab was discontinued due to worsening thrombocytopenia, and the patient was maintained on emapalumab monotherapy for a total duration of 17 months (November 2022–April 2024). Overall, emapalumab was well tolerated. Temporary interruptions in therapy occurred due to nonformulary access and administrative delays, during which transient worsening of inflammatory markers was observed, with subsequent improvement upon resumption of treatment. The patient experienced a transient period of CMV reactivation during therapy requiring interruption of emapalumab and was successfully treated with ganciclovir.

Seven months after the discontinuation of emapalumab, the patient showed no signs of HLH/MAS recurrence (Table 2). The patient remained in remission until November 2025, when she re‐presented with severe cytopenia. As she did not fulfill HLH diagnostic criteria at that time, further evaluation with bone marrow biopsy was performed, revealing myelodysplastic syndrome with excess blasts and monosomy 7 on cytogenetic analysis.

**TABLE 2:** Latest test results of the patient’s HLH parameters.

Parameter	Remission		Second HLH episode	
	At emapalumab discontinuation (April 2024)	7 months after emapalumab discontinuation (November 2024)	November 2025	April 2026
WBC (cells/mm^3^)	4.42	5.9	5.17	6.79
Platelet (cells/mm^3^)	94	191	36	27
Hb (g/dL)	7	8.6	7.7	6.7
Ferritin (μg/L)	3726	654	6085	15,225
IL‐2 receptor (U/mL)	1199	—	1275	1310
Fibrinogen (g/L)	1.42	3.48	3.15	—
AST (U/mL)	23	30	66	53
Triglyceride (mmol/L)	3.4	1.76	1.2	2.85

*Note:* AST: aspartate aminotransferase; Hg: hemoglobin; HLH: hemophagocytic lymphohistiocytosis; IL‐2: interleukin‐2.

Abbreviation: WBC, white blood cell.

During treatment with azacitidine and venetoclax‐based therapy, her clinical course was complicated by febrile neutropenia and septic shock requiring intensive care support, including intubation. This was accompanied by recurrence of HLH, with an H‐score of 205 (88%–98% probability), ferritin levels > 6000 μg/L, and elevated soluble IL‐2 receptor levels (6000 U/mL).

Initial management with GCS and high‐dose anakinra (200 mg daily), administered in conjunction with broad spectrum antibiotics, was not associated with clinical improvement. Emapalumab was therefore reintroduced at a dose of 3 mg/kg twice weekly, resulting in rapid clinical and biochemical response, with subsequent resolution of HLH features and successful weaning from intensive care support.

At the time of last follow‐up (April 2026), the patient remains clinically stable on azacitidine therapy (having completed 3 cycles), with no active HLH manifestations while receiving weekly emapalumab despite persistently elevated ferritin levels (15,225 μg/L).

## 3. Discussion

This case highlights three clinically important and underreported aspects of adult secondary HLH: (1) successful rescue of life‐threatening, refractory disease secondary to SLE with emapalumab, including reversal of stress cardiomyopathy; (2) the safety and efficacy of prolonged IFN‐γ blockade; and (3) effective retreatment of HLH in the context of myelodysplastic syndrome.

First, this report illustrates the therapeutic challenges of managing high‐risk HLH/MAS in adults with underlying SLE. In this patient, the coexistence of active SLE, infection, and recent rituximab exposure contributed to rapid clinical deterioration and diagnostic uncertainty, initially mimicking cytokine release syndrome. Despite timely initiation of HLH‐94–based therapy, treatment was complicated by severe myelosuppression and septicemia, necessitating discontinuation. Such limitations of GCS and etoposide‐based regimens in adults are well recognized and contribute to suboptimal outcomes [6, 7, 16, 17]. Delays in the recognition and management of adult patients with HLH secondary to SLE often result in progression to multiorgan failure and death even with the use of aggressive treatment regimens, as illustrated in a similar case [18]. Similarly, high mortality rates are reported in the context of HLH secondary to lymphoma in Saudi Arabia, despite the use of conventional etoposide‐based therapy [19].

Overall, the suboptimal outcomes with conventional therapies, the life‐threatening nature of HLH, and the considerable variation in the treatment of adult cases illustrate the complexity of relapsed/refractory cases and the need for effective salvage therapy for adult HLH [16, 20]. In this context, emapalumab is recognized as one of the options for use in refractory cases of adults with secondary HLH [21], with both clinical trials and real‐life evidence reporting its safety and efficacy in children and adults [13–15, 22]. Notably, emapalumab can lead to normalization of HLH‐related laboratory parameters and bridging of most patients with severe refractory disease to curative hematopoietic stem cell transplantation [23]. Moreover, recent trials showed rapid improvement following emapalumab use in refractory adult‐onset Still’s disease and systemic juvenile idiopathic arthritis [24, 25].

Consistently, initiation of emapalumab resulted in rapid and sustained clinical improvement in our case, with resolution of hyperinflammation, recovery of cytopenias, and reversal of multiorgan dysfunction. Notably, this included normalization of cardiac function following severe stress cardiomyopathy. While stress cardiomyopathy has been previously described, reported cases have primarily responded to GCS or interleukin‐1 blockade (anakinra) [26]. To our knowledge, this is the first report demonstrating reversal of HLH‐associated stress cardiomyopathy following treatment with emapalumab.

Second, this case provides important insight into the feasibility of prolonged emapalumab therapy. Although emapalumab salvage therapy is conventionally used over shorter treatment durations (8 weeks), the repeated relapse and disease worsening experienced by our patient necessitated the extension of emapalumab treatment for approximately 17 months, with sustained disease control and an acceptable safety profile. Treatment was associated with only limited infectious complications and interruptions in therapy that were followed by biochemical worsening and subsequent improvement upon reinitiation. This prolonged course resulted in a durable remission lasting around 19 months. Real‐world data from the REAL‐HLH study suggest that extended use of emapalumab may be required in selected patients with severe or relapsing disease and can be safely considered, although the overall median treatment duration in this study was 71 days (2.3 months), and the longest duration of emapalumab use did not exceed 11 months in adults [27]. Given that evidence in adult populations remains limited, our findings add to the literature by demonstrating that long‐term IFN‐γ blockade can be both effective and tolerable in complex adult HLH.

Third, this case is notable for the recurrence of HLH in the setting of newly diagnosed myelodysplastic syndrome, representing a distinct and high‐risk trigger of secondary HLH. Malignancy‐associated HLH is associated with particularly poor outcomes and often demonstrates limited responsiveness to conventional therapies [23, 28]. In our patient, HLH recurrence was refractory to GCS and high‐dose anakinra, consistent with prior reports suggesting variable efficacy of interleukin‐1 blockade in this context [29, 30]. Reintroduction of emapalumab, however, resulted in rapid clinical and biochemical improvement, with successful control of HLH despite ongoing hematologic malignancy. This observation is consistent with emerging evidence surrounding emapalumab in the management of malignancy‐associated HLH [31–33], further suggesting that IFN‐γ blockade may retain efficacy across different HLH triggers and disease contexts. Importantly, this case also demonstrates preserved responsiveness to emapalumab upon retreatment following prior prolonged exposure. Data on rechallenge with emapalumab are extremely limited, particularly in adults with secondary HLH. The rapid response observed in this patient supports the absence of apparent resistance to IFN‐γ blockade and highlights its potential role as both salvage and reinduction therapy in recurrent disease.

## 4. Conclusion

This case report contributes to the growing body of literature supporting the use of emapalumab in the management of adult secondary HLH, particularly in relapsed/refractory disease and in patients with underlying autoimmune conditions such as SLE. It demonstrates that emapalumab can achieve rapid control of life‐threatening disease, including reversal of stress cardiomyopathy, and can be used safely over prolonged periods to maintain remission. It also highlights the potential for successful retreatment in recurrent HLH, including in the context of myelodysplastic syndrome. Further research is needed to optimize treatment strategies and improve outcomes for adult HLH patients [34].

**Nomenclature**
AST: Aspartate aminotransferase
CMV: *Cytomegalovirus*
EBV: Epstein–Barr virus
GCSs: Glucocorticosteroids
Hb: Hemoglobin
HLH: Hemophagocytic lymphohistiocytosis
IFN‐γ: Interferon gamma
IL‐2: Interleukin‐2
IV: Intravenous
MAS: Macrophage activation syndrome
SLE: Systemic lupus erythematosus
WBC: White blood cell

## Author Contributions

Hind Salama contributed to the case report by providing, writing, and reviewing all relevant case details. Ghada Alshehri contributed to data collection. Ayel Yahya provided bone marrow analysis. Areej Almugairi contributed in writing the manuscript. Hind Salama had full access to all of the data in this study and takes complete responsibility for the integrity of the data and the accuracy of the data analysis.

## Funding

Sobi provided funding for the medical writing activities and open‐access fees of this manuscript through a medical grant.

## Disclosure

The funders had no role in study design, data collection and analysis, decision to publish, or preparation of the manuscript. All authors have read and approved the final version of the manuscript.

## Ethics Statement

No written consent has been obtained from the patient as there are no patient identifiable data included in this case report/series.

## Conflicts of Interest

The authors declare no conflicts of interest.

## Data Availability

The data that support the findings of this study are available in this article.
